# Safety and efficacy of newer biologics DMARDs in the management of rheumatoid arthritis: A systematic review

**DOI:** 10.1016/j.ocarto.2020.100116

**Published:** 2020-11-01

**Authors:** Basiru Ahmad Zago, A. Priyadharshini, T.M. Vijayakumar

**Affiliations:** Department of Pharmacy Practice, SRM College of Pharmacy, SRM Institute of Science and Technology, Kattankulathur, 603 203, Kanchipuram (Dt), Tamil Nadu., India

**Keywords:** Rheumatoid arthritis, DMARDs, Systematic review, Randomized controlled trials, Efficacy

## Abstract

**Objective:**

To analyze the safety and efficacy of certain biologics DMARDs (Adalimumab, Baricitinib, Pefacitinib and Sirukumab) either used alone or as a combination with MTX for management of rheumatoid arthritis.

**Method:**

We conducted a systematic literature review on various phase 3 Randomized controlled trails, double blind, placebo controlled, parallel group clinical trials for 52 weeks from 2017 to 2019 conforming to the Preferred Reporting Items for Systematic reviews and Meta-Analysis guidelines. The primary efficacy endpoints were American College of Rheumatology 20 response rate improvement criteria, other secondary endpoints were American College of Rheumatology 50/70 response rates, Health Assessment Questionnaire Disability Index, Disease Activity Score-28 for rheumatoid arthritis with Erythrocyte Sedimentation Rate/C Reactive Protein and Radiographic outcomes.

**Results:**

Finally**,** four studies were included for qualitative synthesis in which we observed improvement in ACR 20 response rate was found in the newer agents study group. SB5 (72.4%) at week 24, Baricitinib (70%) at week 12, Pefacitinib 100 mg and 150 mg (57.7% & 74.5%) at week 12 and Sirukumab 50 mg and 100 mg (55% & 54%) at week 16 respectively. ACR 50 and ACR 70 response rate at different point in time was also found to be higher in the study group which indicates their efficacy.

**Conclusion:**

In this systematic review, we observed an improvement in ACR 20 response rate and other secondary efficacy outcomes with an acceptable safety margin. From the evidence of RCTs, we have identified that newer therapeutic agents has beneficial effects when compared to existing therapy.

## 1 Introduction

Rheumatoid arthritis (RA) is a chronic and systemic autoimmune inflammatory disease which primarily affects/alter the lining of synovial joints leading to progressive disability, incapability to participate in social and work activities, increased mortality and also affect the patient quality of life [1]. Rheumatoid arthritis affects both male and female in the ratio of 1:3 most predominantly in females and the onset of the disease can occur irrespective of age but most commonly start at the middle adult age (40–60) years [2]. As per the survey conducted by the Global Burden of disease 2010 “the world wide prevalence of RA is about 0.24% with life time cumulative prevalence approaching ratio 4:2 in female and male [3,4], with 5–50 per 100, 000 newly cases annually [5]. According to the expert opinion on therapeutic patents “rheumatoid arthritis has results in more than 9 million physician visits and more than 250,000 hospitalizations per year in the developed world [6]. In India, about 0.92% of the adult populations were mainly affected by rheumatoid arthritis, thus permanent disability may be prevented with early diagnosis and aggressive therapy [7]. Regardless of recent and tremendous advancing in medical sciences, the treatment of rheumatoid arthritis remains partially effective in the midst of major drawbacks of dose or frequency limiting toxicity [2]. The main purpose/goal of treatment in patients with rheumatoid arthritis (RA) is to achieve remission or at least a lower disease activity, prevention of join damage, disability, and reduced mortality as well as cardiovascular events and other co-morbidities [8]. Currently available drugs either use alone or as combination for treatment of Rheumatoid arthritis include Non-Steroidal Anti-Inflammatory Drugs (NSAIDs), Glucocorticoids (GCs), and Disease-Modifying Antirheumatic Drugs (DMARDs) of both synthetic (conventional like Methotrexate and targeted like JAK- Inhibitors) as well as biological origin (such as TNF Inhibitors, IL-1, IL-6 and B-cell depleting drugs) [9].

Newer biologic DMARDs are considered to be more effective when treatment with conventional DMARD failed in controlling the disease either due to resistance or as a result of intolerable safety margin (level of toxicity) [10]. They have rapid onset of action compared with conventional DMARDs particularly anti TNF. When it comes to the mechanism of action, BDMARDs has defined as well as specific mechanism of action by targeting a single molecule or cell type whereas CDMARD mechanism of action are partly understood [11]. A report of one clinical study indicate that, Biologic DMARDs when given as an induction therapy results in remission rate of up to 80% in patients with RA [12]. Hence, this review focused on the safety and efficacy of certain biologics DMARDs (Adalimumab, Baricitinib, Pefacitinib and Sirukumab) either used alone or as a combination with MTX for management of rheumatoid arthritis (RA).

## 2 Methods

To review/analyze the safety and efficacy of newer therapeutic agents for the management of rheumatoid arthritis (RA), information was extracted from various literatures. For literature screening, different search engines like Clinicaltrials.gov, PubMed, Google scholar and Cochrane library were used and generated a total of 1093 related articles. After removing the duplicates ones, the number were reduced to 760 were we screened them and excluded 736 based on their titles and abstracts. 24 fully texted articles eligible from 2017 to 2019 with Randomized control trials were assessed among which 20 were excluded based on their duration of study either less or exceed 52 weeks. The criteria used for the selection/inclusion of trial were randomized controlled trials (RCTs) conducted for 52 weeks (phase 3). The criteria used for the selection/inclusion of study were phase III, Randomized controlled trials (RCTs), double blind, placebo controlled, parallel group, multicenter study conducted for 52 weeks. Others include patients aged ≥18 years who had an inadequate response to Methotrexate, with presence of active disease defined as ≥ 6 swollen joints, ≥ 6 tender joint (from 66/68 joint count). Finally, four articles were used in the systematic literature review. The keywords used for searching of articles were: Novel therapeutic agents, rheumatoid arthritis and Randomized controlled trials.

For the efficacy outcomes, the study were assessed for at least any of the following improvement criteria: American College of Rheumatology (ACR 20%, ACR 50%, and ACR 70%) response rate; Health assessment Questionnaire - Disability Index (HAQ-DI), Disease Activity Scores for 28-joint counts based on Erythrocyte Sedimentation Rate (DAS28-ESR) or based on C reactive protein (DAS28-CRP) as well as radiographic outcomes. Serious adverse events (SAE's) and mortality (death rate (N) was included for safety outcomes. Sample size was not considered for the selection of the study.

## 3 Results

**Figure 1:**
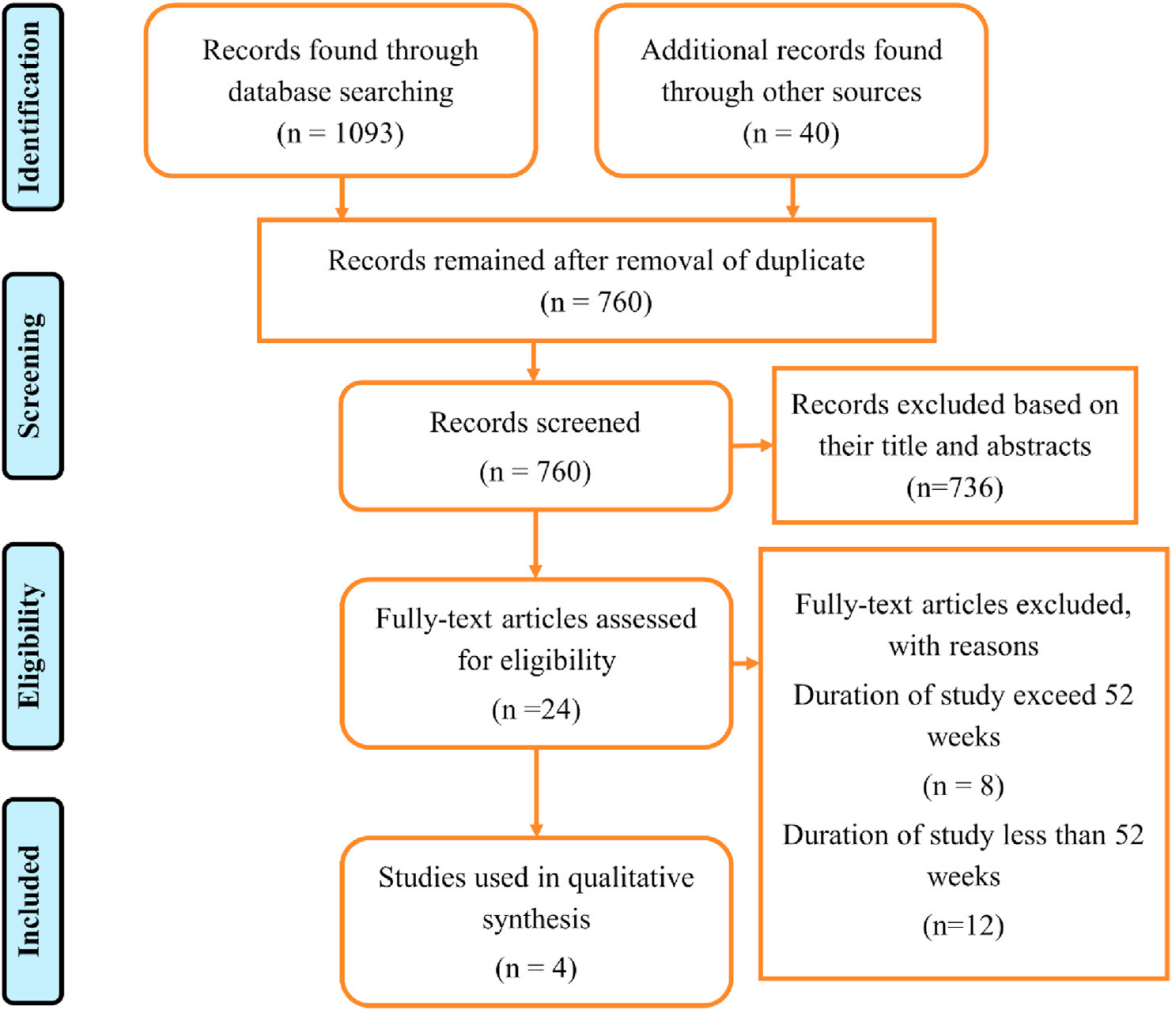
PRISMA flowchart.

Fig. 1 Indicate a PRISMA Flow chart of literature and articles found using different search engines within the clinicaltrials.gov, PubMed, Scopus, Elsevier, and Cochrane library databases. The details of various phase III randomized controlled trials (RCT's) of newer therapeutic agents is shown in (Table 1).

**Table 1:** Details of various Phase III randomized controlled trials (RCTs) of newer therapeutic agents.

Authors	Study Drug	Study Design	Standard Therapy	Study duration	Efficacy Outcome	Safety Outcome (If Any)
Weinblatt ME et al. (2018) [13]	SB5 40 mg SC every other week (n = 239)	Phase 3, randomized, double blind, parallel- group, multicenter study	Adalimumab40 mg SC every other week (n = 237)	52 weeks	Primary end point was ACR 20 response rate and secondary efficacy outcome was ACR 50/70 response rate at 24 week, (DAS28) based on ESR and EULAR response.	Safety outcome includes AEs, SAEs and TEAE's [Nasopharyngitis, Bronchitis, and elevated level of amino transferase, serious infections (Escherichia Urinary tract infection, bronchopneumonia and staphylococcal sepsis). Malignancy].
Taylor PC et al. (2017) [14]	Baricitinib 4 mg OD every other week (n = 487)	Randomized, phase 3, double blind, placebo -and active-control, parallel group trial	Adalimumab 40 mg every other week (n = 330) Placebo (n = 488)	52 weeks	Primary end point was ACR 20 response rate, DAS28-CRP AND HAQ-DI, SDAI at week 12. Major secondary outcome was ACR 50/70; Joint damage progression from baseline at week 24 assessed using mTSS as well as DAS28-ESR.	Safety outcome: Laboratory abnormalities and incidence of severe Adverse events, Serious infection [Herpes zoster Tuberculosis Major adverse cardiovascular events Malignancies Death].
Tanaka Y et al. (2019) [15]	Pefacitinib 100 mg/day (n = 104) Pefacitinib 150 mg/day (n = 102)	Randomized, placebo controlled, double blind, Parallel group phase 3 confirmatory study	Placebo (n = 102)	52 weeks	Efficacy: primary endpoint was ACR 20 response rate. Secondary endpoint was Changes from baseline in 28 joint disease activity score (DAS28-CRP and DAS28-ESR).	Safety: TEAE's [Venous thromboembolism (VTE), Serious infection, Malignancies, Herpes zoster related disease].
Takeuchi T et al. (2017) [16]	Sirukumab 50 mg every 4 weeks (n = 557) Sirukumab 100 mg every 2 weeks (n = 557)	Phase 3, multicenter, randomized, double-blind, placebo- controlled, parallel- group study	Placebo (n = 556)	52 weeks	Efficacy: The co primary efficacy endpoints were ACR20 response at week 16.Major secondary endpoints were change from baseline in Health Assessment Questionnaire– Disability Index (HAQ-DI) score at week 24, ACR50 response rate at week 24, and ACR70 response rate by week 52.	Safety assessments included AEs, SAEs and others. [Increased level in liver enzymes, serious infections (upper respiratory tract infection, bronchitis, and Nasopharyngitis), pruritus, leucopaenia, neutropenia, cardiovascular events, Gastrointestinal (GI) perforations and Death]

SB5 = An Adalimumab Biosimilar; SC = subcutaneous; ACR = American College of Rheumatology (20, 50, and 70); DAS28- ESR = Disease Activity Score for 28 Joint Counts based on Erythrocyte Sedimentation rate; DAS8-CRP = Disease Activity Score for 28 Joint Counts based on C Reactive Protein; EULAR = European League Against Rheumatology; AEs = Adverse Events; SAEs = Serious Adverse Events; TAEs = Treatment Emergence Adverse Events; OD = Once Daily; HAQ-DI = Health Assessment Questionnaire - Disability Index; SDAI = Simplified Disease Activity Index; mTSS = modification of Total Sharp Score; VTE = Venous Thromboembolism.

In a study conducted by Weinblatt M.E et al. the primary efficacy endpoint ACR 20 response rate at week 24 in the SB5 group was (72.4%) which was almost similar in the Adalimumab group (72.2%) with adjusted difference of 0.1% which was within the correspondence margin (±15%). At week 24, ACR 50 and ACR70 response rate between SB5 and Adalimumab was comparable and the proportion of patients whom archived remission based on DAS28-ESR Score <2.6 was higher in the SB5 group compared to Adalimumab group at the same week but the level of significance was not declared. The rate of remission at week 24 for CDAI score <2.8 and CDAI score ≤10 (low disease activity) was also compared between SB5 and Adalimumab group and there was no level of significance declared. Meanwhile the mean changes in scores from baseline to week 24 for both SB5 and Adalimumab groups were compared for SDAI rate of remission and Low disease activity (Table 2).

**Table 2:** Efficacy outcomes of various phase III randomized controlled trials.

Parameters	Weinblatt ME et al. SB5 ADL	Taylor PC et al. Baricitinib ADL P	Tanaka Y et al. Pefacitinib 100 mg 150 mg P	Takeuchi T et al. Sirukumab 50 mg 100 mg P
ACR20	72.4 (w24) 72.2	∗70 (w12) ∗61 40	∗57.7 (w12) ∗74.5 30.7	∗55 (w16) ∗54 26
ACR50	38.1 (w24) 39.7	∗51 (w24) ∗45 19	∗30.8 (w12) ∗42.2 ∗8.9	^º^30.2 (w24) º33.2 12.4
ACR70	19.2 (w24) 20.3	∗30 (w24) ∗22 8	∗13.5 (w12) ∗27.5 1.0	º16.5 (w52) º18.5 5.4
DAS28-ESR < 2.6	21.6 (w24) 19.8	^a^18 (w24)^a^18 5	∗11.7 (w12) е17.8 1.0	- - -
DAS28-ESR ≤ 3.2	34.1 (w24) 35	- - -	- - -	- - -
DAS28 CRP (LSM)	- -	∗-2.24 (w12) ∗-1.95 -0.98	- - -	- - -
DAS28-CRP < 2.6	- -	- - -	∗24.5 (w12) ∗34.7 ∗5	º26 (w24) º25.5 5.6
DAS28-CRP ≤ 3.2	- -	- - -	∗40.2 (w12) ∗53.5 ∗11	- - -
HAQ-DI	- -	∗0.59 (w12) ∗0.54 0.51	- - -	ⁿ0.58 (w24) ⁿ0.57 0.53
LSM changes in SHS	- -	- - -	- - -	∗0.5 (w52) ∗0.46 3.69
CDAI ≤ 2.8	11.3 (w24) 13.6	- - -	8.7 (w12) 9.9 0	!7.0 (w24) !8.4 !3.1
CDAI ≤ 10	29.7 (w24) 29.5	- - -	∗35.0 (w12) ∗54.5 ∗12	∗29.4 (w24) ∗30.2 ∗15.5
SDAI ≤ 3.3	11 (w24) 14.4	∗8 (w12) ∗7 2	8.8 (w12) 8.9 0	- - -
SDAI ≤11	31.1 (w24) 31.1	- - -	∗33.3 (w12) ∗55.4 ∗11	- - -
mTSS	- -	∗0.41 (w24) ∗0.33 0.90	- - -	- - -
SF-36 PCS	- -	- - -	- - -	ⁿ7.74 (w52) ⁿ7.23 6.81
SF-36 MCS	- -	- - -	- - -	ⁿ9.64 (w52) ⁿ9.80 9.57

ACR = American College of Rheumatology (ACR 20%, ACR 50%, and ACR 70% response rates); DAS28-ESR**/**CRP = Disease Activity Scores for 28-joint counts based on Erythrocyte Sedimentation Rate**/**C Reactive Protein; LSM = Least Square Mean; HAQ-DI = Health Assessment Questionnaire-Disability Index; SHS = Sharp/Van der Heijde Score; CDAI = Clinical Disease Activity Index; SDAI = Simplified Disease Activity Index; mTSS = modification of Total Sharp Score; SF-36 PCS/MCS = Short Form-36 Physical Component Summary/Mental Component Summary. ∗*P* < 0.001, ^a^*P* ≤ 0.05, е*P* = 0.003, º*P* ≤ 0.01, ⁿ*P* ≤ 0.001, !*P* ≤ 0.003.

Another RCT by Taylor PC et al. reported that the primary efficacy endpoint ACR 20 response rate for Baricitinib was 70% versus placebo 40% (P < 0.001) which shows a significant response in Baricitinib group compare with Placebo group at week 12. Baricitinib was also compared with Adalimumab at week 12 with a margin of 12% (70% for Baricitinib and 61% for Adalimumab with Confidence interval of 95% between the two groups (2%–15%). As per the statistical analysis, Baricitinib was considered to be better compared to Adalimumab (P = 0.01). Major secondary endpoint results between Baricitinib and Adalimumab were compared and there was a significant improvement in ACR50 and ACR 70 response rate at week 24. Proportion of patients who archived remission based on DAS28-ESR Score of <2.6 in both Baricitinib and Adalimumab group was equal (both 18%) at week 24 (P ≤ 0.05) compared with placebo 5%. Both changes in Health Assessment Questionnaire-Disability Index (HAQ-DI), a remission in SDAI score of 3.3 at week 12 were statistically significant for both Baricitinib and Adalimumab groups (P < 0.001). The least square mean (LSM) changes from base line in structural progression was evaluated at week 24 using van der Heijde modification of the total sharp score and there was a significant reduction in the radiographic progression of structural joint damage for both Baricitinib and Adalimumab group compared with placebo (both P < 0.001 vs placebo). (Table 2).

From the trial of Tanaka Y et al. Compared with placebo, the primary efficacy variable ACR 20 response rate at week 12 for Pefacitinib 100 m, 150 mg and Etanercept response rate were found to be significantly higher with 57.7%, 74.5% 83.5% and 30.7% respectively (P < 0.001). Similarly, at week 12 there was significant improvement in ACR 50 and ACR 70 along with significance increased in DAS28-ESR >2.6 seen in Pefacitinib 150 mg and placebo except for Pefacitinib100mg was not seen owing to complexity in estimating the odd ratio. At the same week the proportion of patients achieved (DAS-28CRP >2.6) was high in both Pefacitinib groups when compared with placebo and were statistically significant (both P < 0.001 vs placebo). The fraction of patients having a CDAI score <2.8 and CDAI score ≤10 (low disease activity) were all higher in both Pefacitinib groups compared with placebo at week 12 with CDAI score ≤ 10 (P < 0.001). The fraction of patients with both SDAI score ≤3.3 and SDAI score ≤11 (low disease activity score at week 12 were significantly higher in both Pefacitinib groups compared with placebo group (both P < 0.001 v. Placebo) for SDAI score ≤11 (Table 2).

In the study of Takeuchi T et al. the proportion of patients achieving ACR 20 response rate at week 16 was significantly higher for both Sirukumab doses (50 mg and 100 mg) (55% and 54%) compared with placebo 26% both (P < 0.001) and was continuous through week 52 irrespective of baseline MTX use. At week 52, the SHS Least square mean changes from baseline in radiographic progression was achieved with differences observed earlier at week 24 in both Sirukumab Doses ((P < 0.001) compare with placebo. For the secondary endpoints significant improvement in both ACR 50 and ACR 70 were reported at week 24 and 52. At week 24, significant improvement based on DAS-28CRP >2.6 was achieved in both doses of Sirukumab and was statistically significant (P ≤ 0.01). The level of significance in HAQ-DI for both Sirukumab doses at week 24 was statistically significance (P ≤ 0.001). The least square mean (LSM) changes from base line in SHS score at week 52 were observed in both Sirukumab group compared with placebo irrespective of baseline MTX use which vividly shows separation between both Sirukumab groups as well as the placebo group (P < 0.001) but not between Sirukumab groups. The proportion of patient who achieved remission at week 24 in CDAI score <2.8 for both Sirukumab group are higher compared with placebo group (both P ≤ 0.003 vs placebo). In the same study the percentage of patients achieving CDAI low disease activity (≤10) at the same week was higher in both Sirukumab groups compared with placebo (both P < 0.001 vs placebo). A significant improvement from changes in baseline in Health related physical and emotional well-being were observed at week 52 with patients reported SF-36 PCS/MCS greater in both Sirukumab group compared with placebo (both P < 0.001 vs placebo). (Table 2).

### 3.1 Safety summary

The serious treatment emergent adverse effect reported was higher in Adalimumab group (8) compared with the SB5 group (3) up to week 24. Among the most common TEAEs reported were Nasopharyngitis, headache, bronchitis, serious infection and none developed an active TB. 2 deaths occurred up to week 24 in the Adalimumab group and it was reported not to be related to the study drug [13]. In the trial of Taylor et al. the rate of serious adverse events through week 24 were reported to be higher (5%) in both Placebo and Baricitinib compared with Adalimumab (2%), 2 deaths were reported in the Baricitinib group [14].In another study, about 7.3% developed serious adverse event in the Pefacitinib 100 mg and 150 mg group and no death were reported throughout the entire study in both the groups [15]. Lastly, serious adverse events were reported in both Sirukumab groups and it was higher in Sirukumab 50 mg every 4 weeks (11%) compared with Sirukumab 100 mg every two weeks (9.8%) through week 52.10 deaths were reported during the entire study and it was as well significantly higher in Sirukumab 50 mg every four weeks (1.1%) compared with Sirukumab 100 mg every two weeks (0.5%) [16] (Table 3)**.**

**Table 3:** Safety data obtained from various phase III randomized controlled trials.

Clinicaltrials.gov Identifier	Study drug	Serious Adverse Events(n)	Death (n)
NCT02167139 [17]	SB5 (n = 239)	3	2
NCT01710358 [18]	Baricitinib (n = 487)	38	2
NCT02308163 [19]	Pefacitinib (n = 206)	15	0
NCT01604343 [20]	Sirukumab (n = 1114)	138	10

## 4 Discussion

The clinical trials we reviewed here highlighted the safety and efficacy of some biological DMARDs (Adalimumab, Baricitinib, Pefacitinib and Sirukumab) either used alone or in combination therapy with MTX leading to reduction in sign and symptoms of RA with significant clinical improvement in physical, emotional health as well as functional status in patients among patients who had an inadequate response to conventional therapies.

In the Clinical study of Weinblatt et al. the ACR 20 response model showed similar efficacy over multiple time points that were assessed during the study at week 24. Moreover, other secondary efficacy endpoint including ACR 50, ACR 70, DAS28-ESR and EULAR response were comparable between the SB5 and Adalimumab groups [13]. Taylor P. C et al. reported that Baricitinib had showed more significant clinical benefits compared with placebo at week 12 in ACR 20 response rate as well as DAS28-CRP. At week 24, there was no observed significant inhibition of radiographic progression in both Baricitinib and Adalimumab compared with placebo and considering the outcome measures as the primary endpoint, the combination of Baricitinib plus Methotrexate was found to be superior compared to Adalimumab plus Methotrexate [14]. Tanaka Y et al. result showed statistical significant in ACR 20, ACR50 and ACR 70 response rate through the first 4–12 weeks for Pefacitinib doses of 100 mg/day and 150 mg/day compared with placebo which were either maintained or improved during the long term treatment for up to week 52. Pefacitinib 150 mg showed greater clinical improvement compared with Pefacitinib 100 mg [15] in the clinical study of Takeuchi T et al. all the clinical efficacy endpoints confirmed that Sirukumab was effective in reducing the sign and symptoms of active RA in a vigorous way. Improvement in ACR 20 response rate in the Sirukumab group occurred as early as 2 week and the response rate was plateaued at week 12 and was maintained all the way through week 52. As early as week 24, there was a significant inhibition radiographic progression with Sirukumab compared with placebo. In this study, the clinical efficacies of both Sirukumab doses were similar [16].

Weinblatt M. E et al. reported that the Safety profile between SB5 and Adalimumab were comparable with similar incidence of TEAE and Serious TEAE and that most of the TEAE were related to the study drug [13]. The study of Taylor P. C et al. reported serious adverse events through week 24 and was found to be more frequent with Baricitinib and placebo compared with Adalimumab. Limitation of this study was that only patients who had an inadequate response to MTX were enrolled there by limiting the capacity to assess the effectiveness of Baricitinib when used in Combination with conventional synthetic DMARDs other than Methotrexate [14]. Safety analysis result of Tanaka Y et al. showed that Pefacitinib was well tolerated for up to 52 weeks. The incidence of TEAE was found to be similar for both Pefacitinib arms compared with placebo with no new safety signal observed. Limitation of this study was that the placebo treatment was shorter duration i.e. 12 weeks compared with Pefacitinib arms thereby limiting the comparison between the two groups. Secondly, radiographic progression was not conducted in the study therefore it was not possible to ascertain whether Pefacitinib inhibit radiographic progression in the study population [15]. Regarding the safety profile of Takeuchi T el al it was consistent with other agents targeting IL-6 and there was no raise in any new safety concerns. The proportion of patients with adverse events (AEs) and serious adverse events (SAEs) were comparatively similar between the treatment groups throughout the study period. The limitation of this study was loss of randomization after the 18 weeks pure placebo controlled period therefore longer total exposure in patient – years to Sirukumab compared to placebo cannot be established beyond 18 weeks which baffle the safety comparison between the two groups [16].

## 5 Conclusion

In the studies we reviewed shown improvement in ACR 20 response rate were seen as earlier as week 12 through week 24 as well as other secondary efficacy outcomes with an acceptable safety margin. From the evidence of RCTs, we have identified that newer therapeutic agents has beneficial effects when compared to existing therapy. However, several studies were uncertain of the long-term effect as an add-on therapy hence; further well designed clinical trials with longer and large sample sizes are warranted.

## Declaration of competing interest

The authors declare no conflicts of interest.
